# A MacIntyrean account of chronic moral injury: Assessing the implications of bad management and marginalized practices at work

**DOI:** 10.3389/fsoc.2022.1019804

**Published:** 2022-10-28

**Authors:** Lily M. Abadal, Garrett W. Potts

**Affiliations:** ^1^Department of Philosophy, University of South Florida, Tampa, FL, United States; ^2^Department of Religious Studies, University of South Florida, Tampa, FL, United States

**Keywords:** moral injury, Alasdair MacIntyre, virtue, manager, management

## Abstract

In this article, we engage with a theory of management advanced by MacIntyrean scholars of business ethics and organization studies to develop an account of “chronic moral injury” in the workplace. In contrast to what we call “acute moral injury,” which focuses on grave, traumatic events, chronic moral injury results from poor institutional form—when an individual desiring excellence must function within a vicious institution that impedes the acquisition of virtues and marginalizes practices. In other words, chronic moral injury occurs when practitioners who pursue excellence in their practice work within corrupt or malformed organizations. To demonstrate this point, we recount the events associated with the rise and fall of the biotech company, Theranos. This case study advances an empirical contribution to MacIntyrean studies by demonstrating how chronic moral injury can happen under such conditions and what the negative consequences may entail for workers.

## Introduction

Research from the field of MacIntyrean Business Ethics has explored the connection between poor management culture and the marginalization of practices (Beadle, 2002; Moore, 2008; Sinnicks, 2018). Considerable effort has been made to resuscitate a positive ideal of management and the manager in Alasdair MacIntyre's terms that would promote practices rather than marginalize them (Beadle, 2013; Moore, 2017; Potts, 2020; Sison and Redín, 2022). In his latest work, *Ethics in the Conflicts of Modernity*, MacIntyre (2016) has spoken approvingly of these hopeful efforts. However, his book nonetheless suggests that many contemporary managers still exhibit disordered desires and problematically view their work through a morally agnostic lens. Too many employees still labor under managers who do not make important matters such as financialization, productivity, and work structure subservient to the intrinsic purpose(s) of the work that their organization exists to promote.

Much of the focus of MacIntyre's critique of contemporary management culture clearly seems to be on the marginalization of practices in the workplace. A broader reading of his research suggests that he is troubled by this phenomenon because of the consequences that exist for individual practitioners who are inevitably formed—for better or for worse—by the work that they do and why they are told to do it. Surveying MacIntyre's research—especially *After Virtue* (MacIntyre, [1981] 2007) and the nuances of his critique that appear in *Ethics in the Conflicts of Modernity* MacIntyre (2016)—we intend to show what he believes these consequences are for employees. By doing so, we seek to jumpstart a discussion about how bad management and the marginalization of practices within organizations impede healthy institutional form and miseducate desires, resulting in a range of consequences—from social to spiritual or existential, psychobehavioral, and so on. *We especially wish to demonstrate how the consequences MacIntyre associates with the social role of the manager can serve as a framework for understanding chronic workplace conditions that may result in Moral Injury (MI)*—“a sense of perceived betrayal unto others, within the self, and/or by an authority figure, that violates personal values and ethics and may result in spiritual, and/or psychobehavioral wounds if reconciliation cannot be achieved” (Richardson et al., 2020, p. 582).

MI is a bio-psycho-social-spiritual syndrome that has gained increasing interest in recent years, beginning with research conducted on military veterans (Koenig, 2018; Hodgson and Carey, 2019; Currier et al., 2020; Richardson et al., 2020; Molendijk, 2021; Chesnut et al., 2022). More recently, scholars have attended to the experience of MI and its connection to higher rates of distress and burnout among healthcare workers (Williams et al., 2020; Mantri et al., 2021). Theoretically, it seems many scholars of MI would agree that personal and disorienting experiences of this phenomenon can happen within any industry or organization. However, very little attention has been given to the subject of MI within the discourse on business ethics, organization studies, and management. This is partly because any adequate identification of MI must first be preceded by a framework or a sensemaking model for conceptualizing how damage to one's conscience as a result of conflict with one's moral compass might occur within a given workplace. We believe that MacIntyre's concerns about contemporary management culture provide precisely the kind of framework that is needed to understand how MI can and indeed often does occur within business organizations.

With this in mind, our article proceeds as follows. First, we briefly sketch a MacIntyrean critique of contemporary management culture and the managerial character. Second, we review work conducted to date on the application of MacIntyre's manager, showing how a positive ideal of management in MacIntyre's terms has been resuscitated—one that promotes practices and healthy, sustainable institutions by placing the highest priority on the intrinsic purpose(s) of the work that is at the heart of an institutions' raison d'être. Third, despite this more hopeful vision of management that aligns with MacIntyre's philosophy, we make a case for the enduring significance of MacIntyre's original archetype of the manager who still lurks in the shadows of MacIntyre's book, *Ethics in the Conflicts of Modernity*. The manager is featured within the context of an account of the changing social roles under capitalism and exhibits a tendency to malform institutions by facilitating the marginalization of practices and the miseducation of desires. We suggest that more needs to be done to conceptualize MacIntyre's enduring concerns about the manager operating under the liberal-individualist conditions of capitalism. Fourth, we review the concept of MI and its effects before introducing a new construct to the existing MI literature. We refer to this construct as “chronic moral injury,” and argue that it is particularly relevant to business ethics and organization studies. As we understand it, chronic MI (CH-MI) results from poor institutional form—when an individual desiring excellence must function within a vicious institution that impedes the acquisition of virtues and marginalizes practices. Like MI more generally, the result of this injury entails considerable spiritual, psychobehavioral, and existential wounds including but not limited to “character loss” (Shay, 2014). The way that we establish this construct relies heavily on MacIntyre's framework, and it highlights the dependency between practices and their respective institutions. Fifth, we make a further case for our addition to the literature by conducting a narrative analysis of CH-MI suffered by the late Ian Gibbons, former Director of Chemistry at the biotech startup, Theranos. As we demonstrate, the very conditions of a contemporary management culture that concern MacIntyre arose within the recent management scandal at Theranos and perpetuated CH-MI, ultimately leading to Gibbons's untimely death. Finally, suggestions for future research on MI within the field of MacIntyrean Business Ethics are made before conclusions are drawn.^1^

## A MacIntyrean account of management in contemporary business organizations

### MacIntyre's critique of modern management culture

Alasdair MacIntyre paints a gloomy picture of the possibility for workplace communities of virtue to exist under the contemporary conditions of capitalism. At one point in the history of human political activity, the Aristotelian ideal of practice-based work cast a vision for tutoring practitioners in the virtues while also advancing what are referred to as common goods—that is, goods that are achieved in common and which serve some prosocial purpose (a well-built house, an excellent car, and so on), as well as those goods of character (the virtues) that are acquired through practices and which contribute to excellence both in practice and in life (MacIntyre, 1953, [1981] 2007). This vision held promise for seeing how the individual's good and the common good might be pursued in tandem *via* deliberation together as communities carrying out a narrative vision of excellence (MacIntyre, 2016, [1981] 2007).

MacIntyre believes older business approaches that once preserved this Aristotelian political ideal could extend the human capacity for virtue cultivation and rational agency *via* active participation within practices that are inherently social and rooted in moral norms bound to workplace traditions. Practices that connect us as members of communities, so MacIntyre's work argues, necessitate virtue cultivation, helping us “learn to recognize, desire, and pursue choice-worthy things” while simultaneously delivering a good or service that contributes to the flourishing of others (Lutz, 2020, p. 27). Now, however, this virtue-informed ideal of practices appears to be marginalized across every imaginable organizational context within the field of business. MacIntyre believes this is the case with very few exceptions.

### MacIntyre's concerns

This section aims to describe MacIntyre's enduring concerns with contemporary business organizations under the current conditions of capitalism. It does so by indicating that his sustained critique, developed over the past 70 years, primarily hinges on the modern character of the manager—“that dominant figure of the contemporary scene” who epitomizes an age where all “organized movement toward power” becomes “bureaucratic and managerial in mode” and all justifications of authority are “couched in terms of instrumental effectiveness” rather than the pursuit of worthwhile projects and choice-worthy things, such as the promotion of human flourishing and the common good (MacIntyre, [1981] 2007, p. 74, 109).

MacIntyre's earliest research into the moral status of contemporary business organizations predates the field of business ethics by nearly three decades. Reflecting on his research that began during the 1950s and 1960s, MacIntyre admits that he was and remains “deeply indebted” to Marx's critical project MacIntyre ([1981] 2007, p. xvi).^2^ He is indebted to “Marx's critique of the economic, social, and cultural order of capitalism,” which underpins his critical narrative about the shift away from practice-based communities of virtue under the order of liberal individualism MacIntyre ([1981] 2007, p. xvi). Understood from the perspective of MacIntyre's corpus, the order of liberal individualism “makes self-interest the only motive for action,” undermining the practice of traditional moral reflection and thus paving the way for the very culture of emotivism that he would come to critique in his most famous work, *After Virtue* (Lutz, 2020, p. 6).

By the time MacIntyre wrote *After Virtue* in 1981, he was settled in this position that liberal individualism has ushered in a culture of emotivism, which diminishes moral judgments to expressions of preference. As Moore (2017) has indicated, “the implication of this is that our society is based on attempts to make others comply with our wishes—“others” become merely a means to our ends, and as a result, all social relations become manipulative” (p. 99). That being said, however, to understand the precise implications of emotivism and the nature of this manipulation in any given context, one must examine the phenomenon in its socially embodied forms. As MacIntyre maintains, “emotivism is a theory embodied in characters who all share the emotivist view of the distinction between rational and non-rational discourse, but who represent the embodiment of that distinction in very different social contexts” MacIntyre ([1981] 2007, p. 30). The form that concerns us here is the archetype of the modern manager, which MacIntyre developed against the backdrop of Max Weber's account of bureaucratic authority.

Indeed, MacIntyre finds that the Weberian managerial archetype is “a conception which presupposes the truth of emotivism” (MacIntyre, [1981] 2007, p. 27). This is because, “on Weber's view, no type of authority can appeal to rational criteria to vindicate itself except that type of bureaucratic authority which appeals precisely to its own effectiveness.” (MacIntyre, [1981] 2007, p. 26). In a culture of emotivism, appeals to morality become nothing more than non-rational appeals to personal preferences. The only appeal that is now viewed as rational for the manager to make in a world characterized by such moral impasse turns out to be an appeal to technique—or to instrumental effectiveness about predetermined ends (MacIntyre, [1981] 2007). MacIntyre believes that this is precisely what has happened in our modern working age. The manager becomes an emotivist character who makes others comply with their wishes by appealing to a “science” of effectiveness. This is problematic for a number of reasons that we will need to briefly explore.

#### The efficacy of a science of management

First, MacIntyre argues that there is no genuine “science” of effectiveness that the manager has at his disposal, and that assertions of any such “science” are simply masking a will to power by using justifications rooted in technique. For something like a “science” of management to exist, the manager would have to possess a “stock of knowledge by means of which organizations and social structures can be molded” to create “law-like generalizations” that would indeed guarantee the manager's effectiveness (MacIntyre, [1981] 2007, p. 77). However, MacIntyre finds that the social sciences come up wanting in this regard, arguing that “the salient fact about those sciences is the absence of any discovery of law-like generalizations whatsoever” (p. 88). In MacIntyre's view, organizations and the practices that they house exhibit too many particular features and variables that preclude the discovery of any law-like generalizations. For example, reducing employee headcount may lead to higher profitability in some business organizations, but this same action could quickly run other businesses into the ground. That a “science” of management is an illusion in MacIntyre's view does not prevent perceptions about its reality from serving the interests of the manager or other executives, however. It is others' belief in this quasi-science mistakenly perceived as genuine rational expertise that legitimizes the manager's authority within the workplace. Moreover, the manager's superiors are licensed to continue prescribing self-serving orders for the manager under the guise of scientific need. Thus, the very appeal to a “science” of management becomes an act of manipulation that masks the manager and other executives' will to power, and MacIntyre (1973) calls readers of his work to “discover the distortions of such an ideology” so that one may avoid, “through self-conscious awareness, being their victims” (p. 337).

#### The efficacy of ends-agnostic instrumentalism

Second, despite there not being a “science” of effectiveness, the fact that the manager's authority is thought to rest upon such grounds means that the business of the manager is understood to hinge on technical efficiency rather than moral goodness. To put the matter more strongly, MacIntyre ([1981] 2007) finds that the manager operates as a character who sees his role through a morally agnostic lens. The manager “treats ends as given, as outside his scope” (MacIntyre, [1981] 2007, p. 30). For this reason, MacIntyre's manager has been referred to as a figure who exhibits “expressions of an instrumental form of reasoning that is concerned with finding the most efficient means by which to achieve given ends but remains unconcerned about the substance of those ends” (Knight, 1998, p. 6). It is not hard to imagine why MacIntyre believes that this is problematic, and the earliest readers of his work did not have to wait for the release of *After Virtue* in 1981 to understand the reasons he provides. In the 1960s, MacIntyre ([1967] 1997) was already developing a critique of management that underscored the problems associated with a character who focuses on instrumentalism while operating from a state of moral agnosticism. He problematized those managers of the Holocaust who “epitomized a purely technical orientation” concerning the efficiency of the Nazi transport and killing machine (Beadle, 2002). Per MacIntyre,

Specialists such as Eichmann…boasted that they merely discharged their function in arranging for so much transport to be provided between points X and Y. Whether the cargo was sheep or Jews, whether points X and Y were farm and butcher's slaughterhouse or ghetto and gas chamber, was no concern of theirs (MacIntyre, [1967] 1997, p. 207–208).

The starkness of this example helps to depict the potentially significant threats to human flourishing and the common good that arise when instrumentalism is exercised based on ends that are prescribed rather than morally justified.

#### The efficacy of profit over purpose

Third, MacIntyre finds that the manager's prioritization of technical efficiency tends to result in the disordered pursuit of external goods over and against goods internal to practices. Swapping the ends (or what MacIntyre calls the “internal goods”) of work for the enhancement of means, the manager places primacy on “external goods” such as “effectiveness in transforming raw materials into products, unskilled labor into skilled labor, and investment into profits” (MacIntyre, [1981] 2007, p. 30). MacIntyre's articulation of the obliteration of ends-thinking and his illumination of means-thinking for the sake of product and profit maximization is intended to illustrate ways that these goods often are mis-prioritized. For an example of what this might look like in practice, consider a manager of a healthcare organization who has been able to effectively increase profitability by cutting time spent with patients in half. It is not hard to imagine, nor is it difficult to assume how such an action—while effectively boosting profitability in the short term—likely leads to reduced patient satisfaction scores and potentially higher rates of misdiagnoses.

MacIntyre's account would be critical of such a move, making it very clear that external goods always ought to be subservient to the more final ends of practices, or the goods internal to productive workplace practices. In the case above, this would mean that financialization ought to remain subservient to the flourishing of patients and those other lives who are either directly involved with or impacted by the practice of medicine. Whenever these priorities are reversed, the managerial language of effectiveness becomes an alienating ‘numbers game’ that tends to exhibit disordered desires which impede virtue cultivation and reduce people and organizational work to their economic value—something that clashes with MacIntyre's Marxist, Aristotelian, and his Thomistic convictions concerning the dignity and needs of human persons as will be discussed further below.

#### The efficacy of work divorced from praxis and moral deliberation

Fourth, the priority shift just mentioned that happens in the modern managerial age is precisely what MacIntyre believes is so corrosive to the Aristotelian notion of virtue-inculcating practices that contribute to human flourishing and serve the common good. Without deliberation about what excellence at work or in other social domains looks like, it is impossible for communities of virtue to survive or retain a narrative vision about what it means to do good work while growing as moral agents. Tragically, because the manager is seen by MacIntyre as the figure most emblematic of a culture of emotivism, the focus on technique for the sake of maximizing whatever ends have been predetermined winds up bleeding into other areas of life beyond the workplace as employees begin to mimic this pattern of reflection that is characteristic of the manager across other areas of their own public and private lives. Everything winds up being “couched in terms of instrumental effectiveness,” or in terms of getting more of what we want without considering whether our wants are in fact “worth wanting” in the first place (MacIntyre, [1981] 2007, p. 77; MacIntyre, 2016, p. 2).

MacIntyre's solution to this problem is thoroughly democratic insofar as he wishes to restore the possibility for all persons to grow in the virtues and contribute to the common good by re-engaging with the Aristotelian ideal of *praxis*. Without participation in practices that situate us in political communities where moral deliberation takes place, MacIntyre does not believe it is possible for individuals to grow in the virtues. What individuals learn through practices is “how to direct their questioning so that they identify correctly—at the level of practice—the ends that they are to pursue as the objects of their desires, learning also how to transform their desires so that they are rightly directed” (MacIntyre, 2016, p. 90). But when practices are marginalized—i.e., when excellence is traded for instrumentalism and the institutions that house practices become corrupted—opportunities for our desires and our character to be tutored in light of the virtues disappear.^3^

Now, because MacIntyre places so much blame on the character of the manager as it pertains to all of this, one of the key questions that has troubled several business ethicists who read *After Virtue* while remaining sympathetic to many of MacIntyre's concerns is whether it might be possible to resuscitate a positive ideal of the manager—one who in fact does promote practices and the common goods derived from them rather than one who contributes to their erosion within the workplace. It is to this matter that we now turn.

### Resuscitating a positive ideal of the manager

As we have just seen, the picture that MacIntyre paints of contemporary management culture and the character of the manager under the conditions of emotivism is far from a positive one, and many business ethicists have leveled critiques against MacIntyre for being too sweeping in his claims. Dobson (1997), for instance, summarizes much of the earliest engagement with MacIntyre's work within the field of business ethics in the 1990s as essentially accusing MacIntyre of being “anti-business” and deploying a “straw-man-type argument” of managerial viciousness to identify what he takes to be an inherent “schism between business and ethics” (p. 126). One scholar who exemplifies Dobson's analysis is Brewer (1997), arguing that in MacIntyre's account one finds a flurry of arguments condemning the manager that are “clouded by his normative bias against the economic sphere” (p. 825). It may be surprising to note, however, that this did not prevent Brewer (1997) and many others who would come later from attempting to square MacIntyre's definition of a practice with a positive ideal of the manager and to do so by attempting to use some of MacIntyre's own statements against him. This section briefly recounts that history, placing particular emphasis on the turn of events that happened largely as a byproduct of conversations beginning with Moore (2002), which have since been picked up by other MacIntyrean business ethicists as well as MacIntyre himself.

#### Early efforts to resuscitate a positive ideal of MacIntyre's manager

Most of the earliest efforts to resuscitate an ideal of management as a practice in MacIntyre's terms, such as Brewer (1997) attempt, have been discredited largely for reasons concerning (a) how they fail to meet the full scope of MacIntyre's definition of a practice, or (b) because they attempt to sneak a “science” of management in through the backdoor, which in one way or another always reinforces the manipulative capitalist conditions of the workplace that MacIntyre criticizes. Useful for our discussion here regarding its various components is (MacIntyre, [1981] 2007) unique definition of a practice, which entails a:

Coherent and complex form of socially established co-operative human activity through which goods internal to that form of activity are realized in the course of trying to achieve those standards of excellence which are appropriate to, and partially definitive of, that form of activity, with the result that human powers to achieve excellence, and human conceptions of the ends and goods involved, are systematically extended (MacIntyre, [1981] 2007, p. 187).

In light of this definition, Brewer (1997) attempt failed, among other reasons, because it promulgated an account of management that does not possess its own internal goods but rather is always conducted for the sake of something else. Moreover, Brewer (1997) attempt to understand management itself as a practice in MacIntyre's terms failed to recognize how the form of activity that management entails is not standardized but instead domain-specific, meaning that the application of wisdom to managing well varies from one organizational context to another (Beabout, 2012). For there to have been any hope for the activities conducted by managers to be construed as a practice, scholars began to realize (a) that it had to be squared with all the features of MacIntyre's precisive definition of a practice, and (b) that it could not entail “an abstract rational method employing supposedly disinterested principles that can be applied to every social domain without regard for the particularities of specific practices and traditions,” as this would effectively entail sneaking a “science” of management in through the backdoor (Beabout, 2012, p. 417).

#### Finding a way forward

As Beabout (2012) recounts in a comprehensive chapter about MacIntyre's influence on business ethics, management, and organization studies, a key turning point in the attempt to resuscitate a positive ideal of the manager in MacIntyre's terms happened when Moore (2002) began searching for a way to understand (MacIntyre, [1981] 2007) account of “the making and sustaining of…*institutions…* [as] a practice which stands in peculiarly close relationship to the exercise of the virtues” (p. 194). Moore (2002) rightly indicated that for MacIntyre, *the making and sustaining of any form of human community is not only a practice in its own right but also a necessary political activity required for the survival of any practices at all*. This is because, per MacIntyre ([1981] 2007), “no practices can survive for any length of time unsustained by institutions” (p. 194).

Now, while MacIntyre ([1981] 2007) work suggests that practices necessarily require institutions for their survival, there is an inherent tension between practices and institutions given that “the ideals and the creativity of the practice are always vulnerable to the acquisitiveness of the institution” (MacIntyre, [1981] 2007, p. 194). In light of this, Moore and Beadle (2006) sought to develop a model of organizational virtue to understand the institutional conditions necessary for practices to flourish and for rational agents' desires to also become rightly directed. Their findings reflected MacIntyre's conviction that unless those managers directly engaged in the making and sustaining of an institution exercise virtues such as “justice, courage, and truthfulness, practices [housed within institutions] could not resist the corrupting power of institutions” in our modern capitalistic age, which tends to celebrate the vice of acquisitiveness as a virtue (MacIntyre, [1981] 2007, p. 194). *Suddenly a positive ideal of the good work that managers could do was beginning to emerge*.

Thus, by exploring what Moore (2002) has famously referred to as “the implications of the practice-institution distinction,” it became clearer within the discourse on business and organization studies what MacIntyre's account suggests about organizations and their need for virtuous management. Moore's scholarship from 2002 to his 2017 book on *Virtue at Work* has proven to be a consistent effort to home in on this project, and it underscores the need for managers who behave virtuously in order to navigate the inherent tension between the needs of institutions and the intrinsic purpose of any organization's “core practice.”^4^ Following MacIntyre, Moore (2008) argues that every organization is characterized by two different types of practices—the “core practice,” or that which marks the institution's raison d'être, and the “secondary practice” of making and sustaining the institution for the sake of preserving its “core” practice. Moore believes that there are significant implications here for the way we ought to think about virtuous management. Specifically, he argues that “management could be redescribed in MacIntyrean terms as the practice of ‘making and sustaining the institution”’ and that managers who attend to this “secondary practice” could enter into “the opportunity to exercise the virtues in pursuit of internal goods…and to be ‘perfected’ in the process” (Moore, 2017, p. 107–108). In summary, the vision that emerged is one of the manager who is necessarily concerned with external goods like profit and an institution's reputation. Importantly, however, the virtuous manager resists the temptation to view such goods as ends in themselves but rather as a means to sustaining an organizational environment where the “core” practice of an institution can flourish along with those agents who participate in that practice.

Once this alternative vision of management was secured, articles from MacIntyrean scholars then shifted in the direction of constructing an account of management informed by virtue that offered narrative visions of excellence within particular domains.^5^. Notably, Beabout (2013) *Character of the Manager* advances a MacIntyrean narrative of management that hinges on the notion of “wise stewardship,” and chief amongst Beabout's virtue-informed theory of management is *phronesis* or practical wisdom. Rather than exercising a subversive “science” of technical effectiveness, this picture of the manager entails one who learns from past successes and failures while striving for *excellence in domain-specific practices*, which requires attending to the unique industry and features of the productive work housed within the manager's institution.

### MacIntyre's response to the resuscitation of a positive managerial ideal

Until recently, MacIntyre mostly ignored the advances made within the field of business ethics that rested upon his account of how managers might also contribute to the common good while growing as rational agents and facilitating a transformation of desires within worthwhile practices. In *Ethics in the Conflicts of Modernity*, however, he signaled approval of those efforts that have sought to reconsider the manager, particularly those which have done so in light of his critiques of capitalism and its liberal individualist tenets. Toward that end, MacIntyre (2016) again emphasized the need for agents “committed to making and sustaining practices through which common goods are achieved,” and he provides further insights into how this might be done virtuously under the prevailing cultural order MacIntyre (2016, p. 110). He argues that agents must “pose political and moral questions about the particular social roles and relationships” they occupy in order to “construct a critique of those roles and relationships” (MacIntyre, 2016, p. 96). By so doing, real-life managers may find that their role consists of desires and prescriptions that are neither conducive to human flourishing nor the common good. In such cases, they “may find some way of disowning the desire” associated with their role that is not found to be ‘worth wanting’ and thus help to “remake the role” in a way that better supports human flourishing and the common good (MacIntyre, 2016, p. 134).

MacIntyre (2016) latest work provides four hopeful accounts of managers who exercise wise stewardship to remake their role in a way that “provides space and resources for [workers]…to pursue ends that they themselves have identified as worthwhile, in the pursuit of which they hold themselves to standards of excellence that they have made their own” (p. 131). In each of these examples, managers resisted the desire to envision “work performed by individuals as never more than a cost-effective means to ends imposed by others for the sake of high productivity and profitability” (MacIntyre, 2016, p. 172). Conscious of their social horizons and moral convictions, such managers instead identify and promote work “because of the common goods served by productive work” and on the basis of what “justice requires” for all those impacted by the practice-based work (MacIntyre, 2016, p. 107). In so doing, MacIntyre argues that those who are resistant to the archetype of the manager from within the social role of the manager will not be principally motivated “by markets and the preferences of those who engage in market relationships” but instead by appeals to work that is “worthwhile” in itself (MacIntyre, 2016, p. 172).

#### *Ethics in the Conflicts of Modernity* and the enduring significance of MacIntyre's managerial archetype

Despite the hopeful examples of management that appear in *Ethics in the Conflicts of Modernity*, it is important for maintaining consistency with MacIntyre that we recognize what he still takes to be the norm in most modern workplaces. Under the conditions of liberal modernity, MacIntyre (2016) argues that the workplace represents a domain where “new forms of oppressive inequality, new types of material and intellectual impoverishment, and new frustrations and misdirections of desire have been recurrently generated” (p. 124). To date, not enough has been said about how *Ethics in the Conflicts of Modernity* can be read as a reflection of MacIntyre's enduring criticisms of capitalism, management, and the guiding liberal “Morality” of preference maximization (what MacIntyre first associates with “emotivism” in *After Virtue* and later with “expressivism” in *Ethics in the Conflicts of Modernity*) that makes it all possible. In reality, we argue that very little about MacIntyre's critique of the Weberian manager has changed since *After Virtue* and that his latest book can be read as an extension of the relevance of this critique, reflected in its most updated and nuanced form.

In other words, it is not to be taken for granted that, since MacIntyre provides hopeful examples of what managers and management *can be*, he has therefore renounced his criticisms of the managerial archetype developed within his earlier work. This archetype persists in his latest book, and his Marxist as well as his Thomistic-NeoAristotelian critiques of the manager and the modern workplace are more pronounced here than ever before. Within this section, and with an eye toward what follows in our discussion about MI, we seek to demonstrate how MacIntyre still believes that the modern conditions of capitalism usher in a culture of employers and other managers who chronically (a) form institutions poorly and (b) miseducate desires MacIntyre (2016, p. 108). We begin with MacIntyre's explanation of how the Weberian managerial type chronically forms institutions poorly.

#### *Ethics in the Conflicts of Modernity* on management and poorly formed institutions

Throughout *Ethics in the Conflicts of Modernity*, MacIntyre (2016) underscores the oft-impoverished nature of those institutions that we lead our lives within under the conditions of capitalism and bureaucratic managerialism. While we have already surveyed what the field of MacIntyrean business ethics has to say about the inherent tension that arises between productive practices and the institutions that house them, MacIntyre's latest book provides new insights for better understanding the conditions under which this tension is poorly navigated by modern managers. MacIntyre maintains that in every negative case, the vision of productive work understood as a service to a common good to which each worker contributes is exchanged for an unreflective commitment to short-term profitability, which is achieved through managerial control without deliberation. This much is not new, however, MacIntyre now contextualizes this enduring critique of the manager's malformed institutional aims in light of Marx's considerations about surplus value, which bring novel insights to the fore about some of the particular components of capitalism that he takes issue with, and thus deepens our understanding of a theory that both ushers in and motivates the character that still concerns him most—the manager.

Following Marx, MacIntyre (2016) maintains that surplus value may be understood as “the value of unrecompensed labor,” which is always beyond what is necessary to sustain an institution, and which gets “appropriated by the employers for their own economic purposes” (p. 97). Surplus value, therefore, serves as the cornerstone of MacIntyre's critique of “capitalist accumulation and capitalist exploitation,” and it is the Weberian manager's goal to exhibit technical effectiveness in securing this surplus value while keeping laborers motivated. So, what *Ethics in the Conflicts of Modernity* reveals about the principle of surplus value is that, on the one hand, it is key to understanding the social role of the manager. On the other hand, it is also crucial for understanding the consequences that this social role inflicts on modern institutions. Among those consequences that MacIntyre associates with poor institutional form are the tendency toward excessive financialization as well as the exploitation of workers, which we shall explore briefly in what follows.

MacIntyre suggests that the managerial principle of surplus value tends to cause institutions to pursue excessive financialization and that it could not possibly do otherwise because the conception of economic activity embodied by this principle inherently turns profit into an end in itself. Just as we have seen elsewhere, MacIntyre (2016) again maintains in his latest work that “it is never permissible to engage in market transactions only to make a profit or primarily to make a profit” (p. 91). Per MacIntyre (2016) account, therefore, managers who exercise the principle of surplus value have already contributed to the malformation of their institution by operating from a paradigm that leaves no room for conceptions of work done principally for the ends of “core” practices and for the common goods that may be derived from them (p. 98).

As it pertains to the matter of exploitation, MacIntyre argues that employers and others who manage an institution that is guided by the capitalist principle of surplus value inherently commodify labor power as well as the workers who conduct that labor, disguising this commodification under the guise of a freely entered contract to work for a designated wage (MacIntyre, 2016, p. 97). They may pursue whatever means are necessary to do this effectively in order to keep workers motivated. For example, they may reinforce the motivations of the working class by touting “cheerful illusions” associated with the capitalist paradigm, perhaps pointing to the satisfactions afforded to the wealthy and powerful, as is so often the case with multi-level marketing pitches or “pyramid schemes,” which tend to begin their video presentations with imagery depicting a house or a luxury sports car that “could be yours too” one day (p. 92). What this tends to lead to on MacIntyre's account is the pursuit of work as a “job,” entered into for the satisfaction of economic desires or other reasons motivated by the principles of self-interest as they are reflected in the reigning “Morality” of individualism. Again here, it is noteworthy to mention that deliberation about ends and common goods has dropped out of the equation.

In summary, what MacIntyre's account of surplus value adds to his picture of bad management is a principle for understanding how institutions become poorly formed with respect to their ends and their social relationships under the conditions of capitalism. Capitalism cannot escape the framework of individual motivation based on self-interest from which it was derived, which always results in these consequences for institutional formation wherever the capitalist principles of self-interest are applied—*that is, unless those who operate from within capitalism do so from an Aristotelian stance of resistance to the spirit of capitalism*. Now, as we shall see next, we not only form institutions but they also form us in MacIntyre's view. What this means is that the manager's failure to form institutions virtuously under the conditions of capitalism, in turn, miseducates the desires of both the manager and the other workers.

#### *Ethics in the Conflicts of Modernity* on management and the miseducation of desires

Another contribution that *Ethics in the Conflicts of Modernity* makes to our understanding of the enduring significance of MacIntyre's managerial archetype has to do with how the aims of the manager under the conditions of capitalism “miseducate” and “wrongly direct” human desires, which inhibits virtue cultivation and human flourishing (MacIntyre, 2016, p. 108). While this conclusion again could have already been drawn based on (MacIntyre, [1981] 2007) previous work, his latest book contextualizes his arguments more clearly in the light of a Distributist critique of capitalism.

From the standpoint of the consumer, what we have to learn from the Distributists about the “paradox of capitalism is that it requires that consumption should serve the ends of expanding production,” thereby “creating consumer societies in which its products can be successfully marketed only if the desires of consumers are directed toward whatever consumable objects the economy needs them to want” (MacIntyre, 2016, p. 109). Managers, therefore, promote an instrumentalist view of advertising that is rife with “seductive rhetoric…which elicits desires for objects that agents, qua rational agents, directed toward the ends of human flourishing, have no good reason to desire” (MacIntyre, 2016, p. 109). Consider, for example, a recent ad released by a nicotine vape company, which suggested that “vaping is 95% less harmful than tobacco!” The seductive rhetoric promises all of the “buzz” with little to none of the consequences, thereby encouraging a habit associated with heart and lung conditions which nonetheless ‘hooks’ millions of new consumers every year.

From the standpoint of the worker as well as the manager, what we have to learn from the Distributists about managers who operate under the individualist principles of capitalism is how they prevent both us and them from exercising practical reasoning in light of what the virtues require to do good work and lead worthwhile lives. This process of becoming tutored in the virtues is disrupted under contemporary management culture because of the manager's ends agnosticism with respect to whatever semblance of a “core” practice remains within workers' institutions (MacIntyre, 2016, p. 108). Thus, drawing on Aquinas, the Distributists remind us that “the point and purpose of monetary exchanges in markets is to enable us all to benefit from each other's labor, and financial gain is to be valued only for the goods for which it can be exchanged and for the needs that it thereby enables us to meet” (p. 91). What this means is that “increasing profit is never a sufficient reason for engaging in one kind of productive activity rather than another or in one kind of market transaction rather than another” (91). Work in the Distributist view involves a conception of achieving common goods, combined with the belief that “it is only in and through the achievement of such goods that individuals are able to achieve their individual goods” (MacIntyre, 2016, p. 92). Such a vision of work helps practitioners recognize and acknowledge their dependence upon others for the goods that they and those others require, thereby promoting a spirit of mutuality and cooperation rather than competition and self-interest.

MacIntyre (2016) contrasts this Thomistic-Distributist vision of just work with the prevailing managerial attitude inherited from thinkers such as Adam Smith, who advocate that the unrestrained individual pursuit of profit will lead in general to increased prosperity for all. This is the standpoint from which the Weberian manager thinks and acts. Modern managers, having no conception of the genuine ends and purposes of work, reinforce the motivation to work for whatever it is that we and they want, which—in a consumer society—tends to be more money to buy more things that we do not really need. Misplaced desire increases consumption as individuals restlessly search for “cheap thrills” and the newest versions of the hottest commodities.

As Lutz (2020) has said about MacIntyre's work—especially his latest book—MacIntyre wishes to provide an account from the standpoint of the everyday person rather than the academic moral theorist about how, practically speaking, we grow as rational agents by “learning to recognize, desire, and pursue choice-worthy things” (p. 27). Unfortunately, however, as we have seen, bureaucratic managers who operate under the individualist tenets of capitalism tend to hamper those institutions and practical activities which do in fact play a role in helping us to grow as rational agents in this regard. Turning now to the literature on MI, we first introduce this concept within the context of the business ethics and organization studies literature and next demonstrate how MacIntyre's critique of the Weberian manager, from his early work to his latest book, serves as a framework for understanding how bad managers and malformed institutions can and do perpetuate MI.

## Envisioning a MacIntyrean framework for moral injury

### What is moral injury?

The concept of MI first entered academic discourse in Shay (1991) with Johnathan Shay's “Learning about combat stress from Homer's *Iliad*.” After many years of working with and studying the traumas specific to veterans, Shay noticed that post-traumatic stress disorder (PTSD) could not fully capture the existential crises many of his patients detailed in therapy. There was more than psychological injury—flashbacks, nightmares, extreme avoidance, hyper-arousal—at play. Shay called this “moral injury.” His insights were later published in a book—*Achilles in Vietnam: Combat Trauma and the Undoing of Character* Shay (1994) where he builds out the concept of MI through the lens of Homer's Iliad. He explains that MI has three components or necessary conditions. First, there must be a “betrayal of what's right” (Shay, 1994, p. vii). In other words, someone must witness an action or receive instruction to perform an action that directly contradicts their values. Second, this action must be done, or the order must be given by someone, who holds “legitimate” or “defined” “authority” (Shay, 1994, p. 6). The perpetrator must be a leader or representative of the institution's values. Finally, the experience must occur in a “high-stakes situation” (Shay, 1994, p. 171). Collectively, this combination amounts to a potentially morally injurious experience (PMIE).

Although Shay's definition was the first, several others have evolved over the past three decades, creating a varied MI landscape in the secondary literature. There have been several notable attempts to provide a comprehensive review of this varied landscape. Hodgson and Carey (2017) pieced together an impressive chronological exploration of the literature beginning with Shay (2002) and ending with Jinkerson (2016). Though they do not present a new definition or suggest that one, in particular, ought to prevail, they contend that “it is important to acknowledge that moral injury is essentially an existential-ontological wound that can have lasting psychological, biological, spiritual, behavioral, and social consequences” (Hodgson and Carey, 2017, p. 1,224). The emphasis in this reminder is certainly on the spiritual; they strongly suggest that further attempts to build upon the existing MI literature would be remiss if they failed to acknowledge the centrality of spirituality in their definitions. In a later article Carey and Hodgson (2018) provide a robust definition that rightly includes this emphasis, which is worth citing at length below:

Moral injury is a trauma related syndrome caused by the physical, psychological, social and spiritual impact of grievous moral transgressions, or violations, of an individual's deeply-held moral beliefs and/or ethical standards due to: (i) an individual perpetrating, failing to prevent, bearing witness to, or learning about inhumane acts which result in the pain, suffering or death of others, and which fundamentally challenges the moral integrity of an individual, organization or community, and/or (ii) the subsequent experience and feelings of utter betrayal of what is right caused by trusted individuals who hold legitimate authority (p. 2).

Note that this understanding of MI includes the legitimate authority caveat present in Shay's definition and a focus on the spiritual dimension of MI.

In 2020, Richarson et al. embarked on a similar project with the aim of producing further clarity on the disjointed definitional history, albeit Hodgson and Carey (2017) were not included in this review. They found that twelve nuanced definitions were consistently cited across military, psychological, and medical journals. Several themes emerged across these definitions which are worth summarizing. First, nearly all conceptions of MI concern “ethics—that is, they reference a moral transgression or violation, using the language of “right” and “wrong” or ethical “beliefs” (i.e., Shay, 1994; Litz et al., 2009; Drescher et al., 2011; Brock and Lettini, 2012; Gray et al., 2012; Stein et al., 2012; Nash and Litz, 2013; Jinkerson, 2016). For example, Brock and Lettini (2012) claim that MI is “a deep sense of transgression including feelings of shame, grief, meaninglessness, and remorse from having violated core moral belief” (p. xiv). Second, following Shay (1994), most definitions utilize the language of betrayal—either interpersonal betrayal or ideological betrayal (i.e., Shay, 1994, 2002, 2011, 2014; Litz et al., 2009; Drescher et al., 2011; Brock and Lettini, 2012; Gray et al., 2012; Stein et al., 2012; Nash and Litz, 2013; Jinkerson, 2016). Intimately tied to this theme of betrayal is the notion of orientation—the source of the betrayal. Many definitions in use mention that MI stems from one of two sources: “(a) one's own perception or meaning of morality and beliefs (i.e., *perception-oriented*), or (b) one's encounter with a potentially morally injurious event (*action-oriented)”* (Richardson et al., 2020, p. 581).

These three themes—ethics, betrayal, and orientation—are the most common features associated with existing definitions of MI. There are, however, additional themes that create varying points of emphasis and nuance to those described above. For example, Shay (1994); Nash and Litz (2013), and Farnsworth et al. (2017) all discuss the inability to reconcile, process, or be virtuous in the wake of MI. Spiritual wounds or existential suffering is mentioned in four of the twelve definitions, bringing to light the connection between MI and the loss of purpose, hope, and meaning (i.e., Gray et al., 2012; Nash and Litz, 2013; Jinkerson, 2016; Farnsworth et al., 2017). A minority of definitions associated MI with the experience of specific emotions such as guilt, shame, and anger (i.e., Brock and Lettini, 2012; Gray et al., 2012; Jinkerson, 2016; Farnsworth et al., 2017). Finally, it is worth noting that Shay (1994) definition is the only one requiring that MI takes place in a “high-stakes” environment, and it is the only definition requiring that a leader must be involved in the betrayal of what is right.

Richardson et al.'s literature review Richardson et al. (2020) demonstrates there is no consensus on how MI should be defined. In fact, since its publication even more definitions have appeared in the secondary literature. However, it seems that the best synthesis of the common themes suggests that “moral injury includes a sense of perceived betrayal unto others, within the self, and/or by an authority figure, that violates personal values and ethics and may result in spiritual, and/or psychobehavioral wounds if reconciliation cannot be achieved” (Richardson et al., 2020, p. 582). We favor this description of MI over Shay's original definition and those who attempted to build upon it through further specification. It is broad enough to include diverse causes and consequences but narrow enough to be meaningful in its application and clearly distinguished from PTSD.^6^ This flexible definition will be important as we apply it to the realm of business ethics.

### Understanding the effects of moral injury

It is beyond the scope of this article to carefully consider each of the “spiritual” and/or “psychobehavioral wounds” that result from MI. However, Shay provides an extended treatment of MI worth summarizing in his analysis of war, which we believe also has implications for business ethics and organization studies. He claims there are two primary effects: the shrinkage of the social horizon and the shrinkage of the moral horizon (Shay, 1994). When a soldier encounters a grave injustice—a betrayal of what is right—in war, their willingness to trust dwindles along with their reverence for the human dignity of their wider community and their comrades. The meaning of previous relationships and commitments fades. It results in “the destruction of the trustworthy social order of the mind” (Shay, 1994, p. 54). Furthermore, the moral horizon of soldiers becomes hazy, if not completely lost. As one of Shay's patients so bluntly puts it, “War changes you, changes you. Strips you, strips you of all your beliefs, your religion, takes your dignity away, you become an animal” (Shay, 1994, p. 56). Without the risk of oversimplification, life seems to lose meaning when damaged by MI. Things that were once important no longer evoke passion. Obviously, this damages a soldier's personal narrative—what did the war *really* mean? Did it mean anything? How should they understand the significance of their life and the difficult choices in it?

Of particular interest to this article is how the total of these effects damage character and thus a person's ability to flourish as a human being through the exercise of the virtues. This reality is also of particular interest to Shay. He boldly states, “the specific nature of catastrophic war experiences […] not only cause lifelong disabling psychiatric symptoms but can ruin good character” (Shay, 1994, p. 31). He further describes in a later publication:

Their ideals, ambitions, and attachments begin to change and shrink. Both flavors of moral injury impair and sometimes destroy the capacity for trust. When social trust is destroyed, it is replaced by the settled expectancy of harm, exploitation, and humiliation from others. With this expectancy, there are few options: strike first; withdraw and isolate oneself from others (i.e., Achilles); or create deceptions, distractions, false identities, and narratives to spoil the aim of what is expected (i.e., Odysseus) (Shay, 2014, p. 186).

Given this description, it is clear how specific psychobehavioral, spiritual or existential wounds can impact the practice of the virtues. Cognitive distortions such as “deceptions, distractions, false identities, and narratives” are substantial impediments to the exercise of practical reasoning (Shay, 2014, p. 186). The destruction of social trust and corresponding shrinkage of the social horizon complicate the exercise of justice. The breakdown of ideals, ambitions, and a sense of meaning frustrates the completion of practices that are directed toward an intelligible *telos*. Moreover, in the absence of purpose, what inspires courage and fortitude? This has led Shay to conclude, in surprising contrast to the American Psychological Association (APA), that even well-formed character can be undone as a result of MI. As Shay notes, “the APA has rejected every diagnostic concept that even hints at the possibility that bad experience in adulthood can damage good character” (Shay, 2014, p. 184). In other words, resistance to this possibility is reflected in the diagnostic manual.

Recently, however, the emphasis on character appears to be receiving more attention in the literature. It is worth noting that Fleming (2022) has distinguished MI with this specific feature—character loss—as complex moral injury (C-MI). As Flemming defines it:

C-MI is the aftermath of non-culpable, world-view discrepant events that disrupt foundational moral beliefs, generating symptoms related to moral disorientation like non-specific guilt, spiritual conflict, demoralization, and a loss of faith, meaning, and hope. Like MI, C-MI is engendered by a moral violation (or moral discrepancy) and experienced as acute moral dissonance. The construct is distinguished, however, by moral violations (or discrepancies) that violate the moral system itself, triggering outcomes associated with a loss of moral function, rather than guilt or blame (Fleming, 2022, p. 1,033).

Though Flemming does not use the language of “character loss” (Shay, 2014) specifically, he does note that C-MI results in an agent's “loss of moral function” (Fleming, 2022, p. 1,033). This denotes the inability to resolve moral dilemmas due to “pervasive, non-culpable guilt, moral confusion, disorientation, spiritual conflict, senselessness, frustration, futility, loss of faith, meaning and hope, and a sense of the absurd” Fleming (2022). In other words, the collapse of someone's moral system strongly correlates with an inability to practically reason—that is, to make ethical decisions. Moreover, Hodgson and Carey (2019) note that when a person suffering from enduring moral injury (E-MI)—the period after the PMIE event when a person is consistently and ongoingly troubled by their moral injurious event—fails to resolve their increasing dissonance, the injury evolves into C-MI.^7^ Ultimately, whether we recognize character loss as a feature of MI more generally or position it as a distinct feature of C-MI specifically, we can gather that MI is an impediment to human flourishing precisely because it darkens and frustrates an individual's moral horizon.

As we prepare to synthesize this concept of MI with MacIntyrean business ethics, there are two important limitations worth noting in the MI literature. First, while interest in MI since Shay (1991) publication has been astounding, leading to new diagnostics, clinical methodologies, and preventative measures, an overwhelming majority of the work has been context-specific, focusing particularly on the development of MI in combat and wartime settings. Even the clinical measures for MI have been developed specifically for the context and circumstances of military veterans and, more recently, healthcare workers, where the language of combatting COVID-19 still creates striking parallels to wartime settings (Koenig, 2018; Mantri et al., 2021). So, while some research has been done to expand the application of the concept to other industries the MI construct has not yet made a significant impact in the realm of business ethics and organization studies. *While blood is not spilled in boardrooms, we hope to demonstrate that there are plenty of settings rife with potentially morally injurious experiences (PMIEs) in capitalist businesses that should incite serious pause*. As we will discover in the following section, MacIntyre's critique of the manager helps us understand how exactly this takes place.

Moreover, the psychological literature tends to focus on what we call “acute moral injury (A-MI).” We consider it acute because it assumes a conception of MI that depends upon extreme, obvious, and grave experiences or events. Such experiences are easy to identify and focus on because they are so obviously problematic. Wartime traumas are intense traumas. Imagine witnessing the murder of a civilian or the torture of a prisoner of war. Even worse, imagine being ordered to do these things by a commanding officer. To use Arendt (2006) language, these moral ills are “fanatically” evil (p. 26). We argue that MacIntyre can help us understand another sort of MI that results from a slow and steady betrayal of what is right—poor institutional form. We call this “chronic moral injury (CH-MI),” to clearly contrast this type with the A-MI that garners myopic attention in the secondary literature. It will be our goal in the following section to tease out these claims and suggest why they are important topics for further research and consideration.

### MacIntyre's managerial archetype and chronic moral injury?

We spent the preceding sections discussing the definition of MI and its side effects. To review, MI can be defined as “a sense of perceived betrayal unto others, within the self, and/or by an authority figure, that violates personal values and ethics and may result in spiritual and/or psychobehavioral wounds if reconciliation cannot be achieved” (Richardson et al., 2020, p. 582). Thus, MI is a specific sort of moral trauma with a wide array of consequences impacting multiple dimensions of the human person and which can collectively produce what might be described as character loss or impairment. We will now use MacIntyre's framework to make sense of what we call CH-MI. As we will come to show, CH-MI occurs when institutions betray the internal and common goods they must strive to promote and therefore marginalize the very practices they aim to sustain. This sort of poor institutional form is perpetuated by the navel-gazing, bad manager. Thus, MacIntyre helps us understand and frame a novel conception of MI that demands the attention of psychologists, business ethicists, and scholars of organization studies. Now, in order to build out this concept of CH-MI, let us first recall what we mean here by “poor institutional form” using MacIntyre's language and framework as a guide. Afterward, we will turn our attention to explaining how MI results from it.

MacIntyre's framework suggests that practices are necessary for the cultivation of virtue and that institutions are necessary for the continuation of practices. The purpose of institutions is to sustain and promote the excellence of practices—for the sake of their internal and common goods. MacIntyre identifies a constant tension between institutional demands and the purity of practices where the pursuit of external goods dominates. The mark of a good institution is one that can balance intuitional demands without sacrificing the excellence of the “core” practice(s) it exists to promote. If there is a lack of concern for institutional needs, practices cannot be sustained because the institutional resources needed to sustain them are jeopardized. If there is an excess of concern for institutional needs, practices become corrupted and stand in need of moral criticism; practitioners are reduced to technocrats that embody skills to sustain a shadow of some pre-existing mission or *telos*. We can refer to this process—an excess of concern for institutional needs and external goods at the expense of sustaining the excellence of “ore” practices—as the marginalization of practices in the workplace. We may call an institution with such poor form a vicious institution, as it no longer promotes those practices which are essential to the formation of virtue, but instead promotes efficient, technical skill that becomes detached from good and worthy ends (Moore, 2012b).

As we demonstrated in Section A MacIntyrean account of management in contemporary business organizations, this process—the marginalization of practices—is sustained and perpetuated by the Weberian manager operating under the individualist principles of capitalism. Such a manager deliberately seeks technical effectiveness in the procurement of surplus value—that which is over and above the pursuit of worthwhile institutional needs. It is here, working for the Weberian manager, that we locate our victims of CH-MI. *Bringing all this together, CH-MI occurs when practitioners who pursue excellence according to the standards of their practice work within organizations where vicious performance is normative and expected*. Such organizations are those that have succeeded in marginalizing practices and deprioritizing the internal and common goods that supposedly define the organizational *telos*. As such, CH-MI is banal, bureaucratic, and perpetuated by the Weberian manager that MacIntyre vigorously critiques. Thus, there is a slower and less-noticeable onset of injury that persists for longer periods of time. Like a physical chronic injury, a moral chronic injury may not result from one particularly intense event. Instead, it is a slow and steady betrayal of what is right, resulting from poor institutional form—a vicious institution.

Now, the manager within such a vicious institution demands one of two things from the practitioner pursuing excellence in their practice: (a) complete assimilation to the standards of technical effectiveness, or (b) silent protest. Both of these expectations are a form of CH-MI. Both involve a betrayal of what is right. Both complicate the individual's sense of meaning and purpose. Both have significant social, spiritual or existential, and psychobehavioral consequences. In the first case, the worker is being asked to no longer desire the goods internal to their practice. In effect, the employer attempts to miseducate the worker's desire by encouraging mere bureaucratic effectiveness at the expense of seeking good and worthy ends. Imagine a lawyer who practices law but does not work for justice, a doctor who practices medicine but is not chiefly concerned with the good of health, or a chemist being asked to abandon the scientific method and misrepresent data. This is, no doubt, a form of character loss. The institution demands vice and the worker complies for the sake of their livelihood and, perhaps, the love of the goods internal to a practice they can no longer exercise with integrity.

In the second case, the worker is asked to be in the institution but not of it—to work within it and not be formed by it. This call to compartmentalize deeply held values and beliefs is, of course, an unrealistic expectation of secularism. Even if the worker has not forsaken the excellent standards of their practice, they may suffer from other forms of virtue loss. Perhaps they grow increasingly angry and bitter, which distorts their ability to practically reason and enjoy their work. Maybe the scope of their social horizon shrinks as they lose trust in those managers who are in places of authority over them. They are, effectively, being asked to suffer long bouts of cognitive dissonance, and this complicates the meaning and narrative unity of their lives. In both of these instances, assimilation and silent protest, the marginalization of practices causes CH-MI when workers actually desire or seek excellence in their practices. In other words, we could not speak of CH-MI as we have so far defined it among workers who do not seek excellence in their specific practices. It would be possible to ascribe some instances of acute MI to such workers but not CH-MI because the latter suggests that a worker's necessary institutional affiliation impedes the acquisition of virtues that are otherwise made possible through excellent praxis.

It is important to recognize that there is a spectrum of possible conditions that could yield an experience of CH-MI, ultimately forcing assimilation to the standards of technical effectiveness or silent protest as well as the resulting character loss that comes with it. For instance, some workers are financially vulnerable and unable to find work outside their current place of employment. They may sincerely need the paycheck in order to pay their mortgage, provide for their children, or meet other basic needs. Moreover, perhaps they are dependent upon other benefits available to them through their employer. Health insurance is a good example of this, especially in cases where workers are physically ill, have some disability, or could become pregnant. In other words, low socioeconomic status may put one at an increased risk of experiencing CH-MI. Another possibility is that workers may be highly specialized and have few options available to them where they can engage in their workplace practice of choice. A job change in this instance may take a longer time or require one to abandon the specialization that they have spent years cultivating. MacIntyre's insistence that practices depend upon institutions is particularly poignant here. Finally, we can imagine a situation in which a worker has tried to engage with management about the lack of ability to achieve excellence in their practice to no avail, lacking the influence or position of power to make any substantial change. This, of course, could mean they may have the courage to fully confront the dissonance that they are experiencing with management but are still dependent upon their institution for some reason. Suffice it to say that a worker's ability to simply exit a situation in which they are being forced into assimilation or silent protest is more complicated than we may initially imagine, especially given the fact that practices are institutionally dependent. Sartre may call this “bad faith” (Sartre, [1947] 2007, p. 47) but Sartre also had the privilege of a bourgeois upbringing and did not recognize that human beings are dependent rational animals (MacIntyre, 1999).

We hope it is now clear how CH-MI is the result of poor institutional form. Now, it is worth addressing how *this* sort of MI differs from that which has been discussed in the existing MI literature. Acute MI can be described as an intense event that betrays an individual's sense of what is right. In the context of work, we can think of acute MI as the sort of thing that would be handled by human resources. They are actions—or isolated events—that are clear moral violations and not necessarily tethered to the structure of the institutions in which they take part, nor do they concern the nature of the work people perform. It happens when a respected leader is found guilty of sexual assault. It happens when they discover that a male coworker with less experience and the same job title has a significantly higher salary. It is lying about a co-worker's performance to obtain a promotion over them. These are actions and events of individuals that violate expectations of fairness, justice, or deeply held moral beliefs that are difficult to reconcile with the narrative of one's life. They are clear betrayals of what is right. If severe enough, they have significant social, psychobehavioral, and spiritual-existential consequences, and such betrayals can constrain the exercise of virtue. How does an individual internalize that relationship with their former mentor who is now ousted as a predatory administrator? How do they understand their dedication to a company that doesn't seem to value their contributions because of their gender? If you were a worker at a just institution, these acute experiences, though injurious and painful, would be handled accordingly—if and when they are found out. The expectation would be that the predatory administrator would be fired, for example. They are, in fact, violations of some policy discussed at some training or orientation held by an institution, which are typically tethered to principles derived from deontological ethical theory.

**Figure 1:**
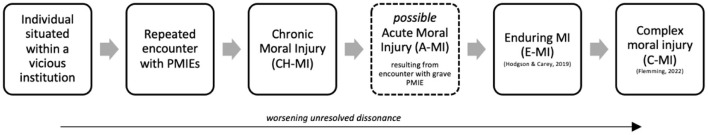
A progression of chronic moral injury (CH-MI).

Thus, the difference between acute and CH-MI is that CH-MI specifically relates to the form of an institution and how that form deprives individuals of seeking the goods internal to practices that are necessary for promoting human flourishing and the common good. Vice becomes an expectation and the goods internal to the pursuit of excellence in the practice become thwarted. We hope that this can be clarified in Figure 1 below. This figure demonstrates the concept of CH-MI from onset to the potential development of C-MI within the context of a corrupt or vicious institution. This would include, but not be limited to, business organizations. As shown in the figure, CH-MI occurs within the context of a corrupt or vicious institution. Over repeated exposure to potentially morally injurious experiences (PMIEs), the individual develops CH-MI which can subsequently develop into E-MI and eventually C-MI if the individual's increasing dissonance is not resolved. Thus, CH-MI has the same consequences as MI more generally.^8^ It is possible for an acute experience of MI to occur within a chronically morally injurious context, which could compound MI and worsen side effects. Though a potentially morally injurious experience must necessarily precede CH-MI, we contend that an acute injury does not; CH-MI is not necessarily the accumulation of many intense, acute injuries over time.

We have been able to make this distinction between A-MI and CH-MI through the application of MacIntyre's Thomistic-NeoAristotelian theory of the virtues and his corresponding Marxist critique of the Weberian manager. As a result, we have provided a framework for conceptualizing the effects of poor institutional form that can be applied across industries spanning the fields of business ethics and organization studies. Now, in what follows, we will turn our attention to a case study on CH-MI in order to better elucidate this distinction.

## Discussion: Theranos as a case example of bad management and CH-MI

Many in organization studies and business ethics will be familiar with the rise and fall of Theranos—a biotech company started by Elizabeth Holmes that hit a 9-billion-dollar valuation just before leadership was indicted on charges of fraud. The story is one that obviously demonstrates several important lessons regarding deception in Silicon Valley, transparency in leadership, and the corrupting power of greed. We believe there is also a story of CH-MI here^9^. Though there are dozens of individual cases we could explore, the most glaring is that of Ian Gibbons, the late biochemist and chief scientist at Theranos. We argue that Gibbons was a *good* chemist. In striving to maintain the standards of his practice within a vicious institution, he met his untimely death. As we trace the Theranos story, it is worth noting that we will be primarily using the reporting and narrative of Carreyrou (2018) at The Wall Street Journal. Carreyrou published dozens of articles on Theranos between 2014 and 2019, including the original exposé that ousted the company. He is also the author of *Bad Blood: Secrets and Lies in a Silicon Valley Startup* Carreyrou (2018), which presents the entire Theranos story from inception to collapse.

The idea for Theranos began with CEO and founder, Elizabeth Holmes in 2003. She dropped out of Stanford to pursue the dream of revolutionizing healthcare by using small fingerpricks of blood to run hundreds of diagnostic tests on a portable machine. By 2010, Holmes was able to secure nearly 100 million dollars in venture capital and built an impressive board of directors. She was able to do so because she presented an inspiring mission. Her product would, she claimed, make preventive care affordable and accessible to the masses. Holmes championed Theranos in this way—as an agent for health equity. As she explained in an interview with Bill Clinton,

Our work is in the belief that the access to healthcare information is a basic human right. […] Health data needs to be accessible to people before they get sick. […] Our work is in being able to make lab testing accessible in time for therapy to be effected and to do that in a way where every person irrespective of their insurance status, irrespective of where they live, can afford the ability to get a test done (Holmes, 2015).

Theranos supposedly existed for this purpose—to expand access to needed health care. Though this purpose was communicated in interviews and pitches, Theranos was never actually a company that principally sought to expand access to health. In truth, Holmes had a very different mission—to be a billionaire (Carreyrou, 2018, p. 11). This is where the vicious formation of the institution began—at the beginning and with the wrong *end*. She sought exponential growth over and above actually developing an innovative product in biotechnology that would contribute to human flourishing and the common good.

This end—becoming a billionaire—colored the way Holmes led and managed everything at Theranos. This is, after all, the usefulness of final ends (Frankfurt, 1999). They inform, guide, and direct our actions, and they do so regardless of whether the appropriate final ends are pursued. Holmes made extraordinary claims about the capabilities and possibilities of the technology that could not be supported by her chemists or engineers. She carefully created silos to ensure that employees did not understand the full scope of the project's development. She held outrageous expectations for her workers, sometimes demanding departments to run 24/7. She treated employees as disposable linchpins, quickly discarding those who did not fall in line. Even worse, she would punish those who challenged the morality of her actions by firing them, demoting them, or ceasing to give them pertinent information. “She was so laser-focused on achieving her goals that she seemed oblivious to the practical implications of her decisions” (Carreyrou, 2018, p. 26). Indeed, Holmes would pursue whatever means necessary to make her billionaire vision a reality. Vice was not merely excused. It was expected.

Moreover, Holmes's myopic billionaire vision effectively marginalized the two “core” practices vital to the success of her project—biochemistry and engineering. She hired excellent scientists who were trained at Cambridge and Ivy League institutions. However, Holmes did not want them to be excellent scientists. She wanted technocrats to create her product for the sake of maximizing profitability. Holmes insisted that “the Edison”—the machine that Theranos was working on to conduct the blood tests—had to be portable and had to use only a small droplet of blood. She was uncompromising in these demands because she already promised them to investors. She did this without really understanding whether they were scientifically possible. When the evidence repeatedly showed that more than a droplet of blood was needed to conduct the tests, she barreled forward with more vigor. It was as if she hoped to yield a successful test by sheer force of will. When her teams did not produce the desired answers, she shrouded their results in secrecy. She kept moving things forward—scheduling clinical trials and seeking partnerships with drug stores—though the technology was not ready or working accurately. Holmes settled for the guise of success in the lab, not excellent science.

It was within this highly vicious structure at Theranos that Ian Gibbons worked as the Director of Chemistry. Carreyrou (2018) develops an exceptional character sketch of Gibbons during his time at Theranos, explaining that Gibbons was known for his dedication to and love of his craft (p. 281). “He was passionate about the science of blood testing and loved to teach it. In the company's early years, he would sometimes hold little lectures to educate the rest of the staff about the fundamentals of biochemistry” (Carreyrou, 2018, p. 281). Most importantly, Gibbons had high standards of excellence. He was uncompromising in not making concessions or cutting corners that would somehow endanger the veracity of test results and subordinate concern for patients to the product itself. He was tireless in his conviction that things “must be done right” (Carreyrou, 2018, p. 202). Gibbons was, in fact, a chemist who pursued excellence in his practice, and he was beloved by his coworkers and his dear wife.

As a result, many things at Theranos troubled him. He was open about his frustrations with Holmes's management, particularly the intentional silos she created, the secrecy, and the exaggerations about the technology's readiness. He expressed frustration to a co-worker when Theranos moved forward with pursuing a partnership with Walgreens, implementing Theranos clinics at their neighborhood pharmacies. The technology was not ready; it did not work. Gibbons's willingness to vocalize the truth about the science threatened the secrecy Holmes worked hard to maintain. She did not want his expert opinion, even though his job was to do precisely that—to provide his expert opinion. When Holmes caught wind that Gibbons was discussing these realities with coworkers, she attempted to fire him. However, his expertise proved invaluable, and he was offered another role at the company without the same access to information and authority in his craft. He took the demotion, hoping he could continue doing what he loved in some worthwhile capacity.

When Gibbons was re-hired and demoted at Theranos, he was effectively silenced and removed from the lab. He started working from home as a mere technical consultant, remaining steadfast in his commitment to excellence. The new Director of Chemistry was much more willing to compromise in places where Gibbons would not relent. However, Gibbons was still committed to the task, arguing for hours over the phone about the importance of accurate testing and how anything less than excellent results would negatively impact patient wellbeing. His unwavering pursuit of standards rendered him a defunct chemist at a company he knew to be deceitful.

The circumstances were horrible, so why didn't he just leave? At this point, Gibbons was 67 years old, he had recently been diagnosed with cancer, and he was battling undiagnosed depression. As Carreyrou (2018) explains, he felt it was unlikely that he would be able to secure another job as a chemist if he left Theranos (p. 204). Things just kept getting worse from here for Gibbons. He was called to a deposition in a case regarding several Theranos patents, and this forced him into a corner. He had two choices: lie and perjure himself about the nature and origin of the patents, or break an NDA with Theranos and face Holmes's wrath. It was a heavy moral and existential predicament, and Gibbons saw no way out. Theranos attorneys kept pressuring him to lie, to push back the deposition, to use his cancer as an excuse to evade court, and so on. But this did not mesh with Gibbons's character. He was not a liar, but if he were to go on, he also needed a job and health insurance. This acute MI at the end of his long chronic struggle at Theranos weighed on Gibbons heavily. Linking this instance to Figure 1, it is worth emphasizing here that this is a case of experiencing CH-MI and A-MI in tandem. In the midst of this painful dilemma, Gibbons tragically took his own life. Theranos leadership barely spoke of it after he had been with the company for 10 years and produced most of the patents that they had secured. Rather than conveying her remorse to his wife, Holmes told her that Gibbons's laptop and corporate documents belonged to Theranos and that she needed to return them to the company promptly.

In looking at the story of Ian Gibbons and his time at Theranos, it is easy to see the marginalization of practices on display. Holmes vigorously pursued a billion-dollar valuation at the expense of scientific integrity. She hired particularly excellent scientists and demanded that they sacrifice their commitment to excellence for the sake of Theranos and its feigned success. Chemists and engineers at Theranos were expected to betray the standards of the scientific community. In fact, they were expected to not care about them at all. One could argue that Gibbons was so deeply habituated as an excellent chemist that he could not simply betray those standards for Theranos. His virtuous desires could not be deformed by the Weberian manager. Instead, during his last few years at Theranos, Gibbons was forced into a position of silent protest, enduring the stress of significant cognitive dissonance. There is a particular sort of pain when someone like Gibbons—a practitioner committed to excellence in his practice—is pressured to comply with vice as a worker in a poorly formed institution. We describe this pain as CH-MI.

It is easy look at this case study and draw the conclusion that such circumstances are rare—to hold up the example of Elizabeth Holmes as a fanatical egomaniac. Likewise, it is easy to hold up the example of Theranos as an exceptionally disingenuous start-up. Following MacIntyre's critique, perhaps this isn't so. Perhaps there are more lawyers being thwarted the opportunity to work for justice, doctors being thwarted the opportunity to work for health, and chemists being thwarted the opportunity to uphold the standards of scientific method than we are comfortable knowing. Holmes's persona and the structure she implemented at Theranos were, after all, openly borrowed from Steve Jobs. She captivated Silicon Valley because she was familiar—tenacious, cold, and obsessed with success. The secrecy and results-driven demands that characterized the Theranos culture were easily dismissed and excused because of how closely they mirrored the norm. Thus, we conclude that the setting ripe for CH-MI is common and pervasive.

## Conclusion

In this article, we have demonstrated how MacIntyre's framework can be used to more deeply understand MI through the development of a new concept—“chronic moral injury.” In contrast to what we call “acute moral injury,” which focuses on grave, traumatic events, CH-MI results from working under a vicious manager and/or within a vicious institution. Thus, CH-MI provides language to describe the real anguish of a worker unable to pursue genuine excellence. Moreover, it points toward the institutional dependency workers have on their managers and their place of employment, not just for a paycheck, but to flourish as human beings and pursue the common good. Most importantly, we hope the language of CH-MI and its location within the larger MI context (as depicted in Figure 1) empowers those working in such traumatic conditions to better narrate and understand the source of their pain and inner conflict.

While MI has been taken seriously in the military literature and more recently in the healthcare field, it has not received due attention in the fields of business ethics and organization studies. There is a need for more research examining this phenomenon of MI, particularly CH-MI in the workplace. MacIntyre's critique of the manager and his insights into the practice/institution dependency are essential for understanding this conceptually, as we have outlined in this article. Building on the work already established by MacIntyrean business ethicists, the fields of business ethics and organization studies should continue to carefully consider how practices and their corresponding standards of excellence can remain primary in a capitalist business context. As we have shown, the stakes are high. There is also a need for further empirical, psychological research to demonstrate the relationship between poor institutional form and MI, taking seriously the dependency between the practices needed to cultivate virtue and good institutions. More specifically, there may be a strong correlation between experiences of acute MI within chronically morally injurious contexts that merits further exploration. Finally, in the scope of this article, we have not been able to delve deeply into the specific harms that result from CH-MI, beyond gesturing toward the fact that they are in many ways analogous to those identified by military researchers. Thus, we believe that more empirical work is needed to assess the relevance and further implications of MI in other institutional domains, and we hope that we have inspired MacIntyrean business ethicists and scholars of MI to work together toward this end.

## Data availability statement

The original contributions presented in the study are included in the article/supplementary material, further inquiries can be directed to the corresponding author/s.

## Author contributions

LA and GP conceptualized this article together. GP focused primarily on the MacIntyrean underpinnings of the argument and LA developed a new concept called chronic moral injury to describe its implications for business ethics and organization studies. All authors contributed to the article and approved the submitted version.

## Conflict of interest

The authors declare that the research was conducted in the absence of any commercial or financial relationships that could be construed as a potential conflict of interest.

## Publisher's note

All claims expressed in this article are solely those of the authors and do not necessarily represent those of their affiliated organizations, or those of the publisher, the editors and the reviewers. Any product that may be evaluated in this article, or claim that may be made by its manufacturer, is not guaranteed or endorsed by the publisher.

## Footnotes

^1^We are extremely grateful to the editor of this special edition, Geoff Moore, and the two reviewers who offered invaluable suggestions during the revision process. The final version of this article is indebted to them and their thoughtful comments.

^2^In particular, see MacIntyre (1953, 1958, 1959, [1967] 1997).

^3^For more on this, see Sinnicks (2018).

^4^Other noteworthy papers by this business and organizational scholar that further developed this framework for virtuous management include Moore (2005a,b); Moore (2012a,b); Moore (2015, 2016).

^5^See, for example, Moore (2012b); Beadle (2013); Robson (2015), as well as Bernacchio and Couch (2015).

^6^For more on the distinction between MI and PTSD, see Potts and Abadal (2023).

^7^Hodgson and Carey (2019) defined E-MI at a meeting of The Australian Military Medicine Association in 2019.

^8^It is worth noting that because Fleming (2022) published his findings on C-MI prior to this concept of CH-MI surfacing in the literature, he notes that C-MI stems only from acute experiences of MI. Thus, this figure can be read as a suggested revision to Flemming's recent research insofar as we associate C-MI with both A-MI and CH-MI.

^9^Since all of the information about this case is available in the public domain, the name of the company and the names of the individuals involved have not been made anonymous.

## References

[B1] Arendt A. (2006). Eichmann in Jerusalem: A Report on the Banality of Evil. London: Penguin Books.

[B2] Beabout G. (2012). Management as a domain-relative practice that requires and develops practical wisdom. Business Ethics Quart. 22, 405–432. 10.5840/beq201222214

[B3] Beabout G. (2013). The Character of the Manager. Basingstoke: Palgrave Macmillan. 10.1057/9781137304063

[B4] Beadle R. (2002). The misappropriation of MacIntyre. Philos. Manag. 2, 45–54. 10.5840/pom2002226

[B5] Beadle R. (2013). Managerial work in a practice-embodying institution: the role of calling, the virtue of constancy. J. Business Ethics 113, 679–690. 10.1007/s10551-013-1678-2

[B6] Bernacchio and Couch (2015). The virtue of participatory governance: a MacIntyrean alternative to shareholder maximization theory. Business Ethics Eur. Rev. 24, 130–143. 10.1111/beer.12101

[B7] Brewer K. B. (1997). Management as a practice: a response to Alasdair MacIntyre. J. Business Ethics 16, 825–833. 10.1023/A:1017997200200

[B8] Brock R. N. Lettini G. (2012). Soul Repair: Recovering From Moral Injury After War. Boston, MA: Beacon Press.

[B9] Carey L. B. Hodgson T. J. (2018). Chaplaincy, spiritual care and moral injury: considerations regarding screening and treatment. Front. Psychiatry 9, 619. 10.3389/fpsyt.2018.00619 30568605 PMC6290645

[B10] Carreyrou J. (2018). Bad Blood: Secrets and Lies in a Silicon Valley Startup. New York, NY: Knopf.

[B11] Chesnut R. P. Richardson C. B. Morgan N. R. Bleser J. A. Mccarthy K. J. Perkins D. F. (2022). The moral injury symptoms scale-military version-short form: further scale validation in a U.S. veteran sample. J. Relig. Health. 61, 3384–3401. 10.1007/s10943-022-01606-5 35790578

[B12] Currier J. M. Isaak S. L. McDermott R. C. (2020). Validation of the expressions of moral injury scale-military version-short form. Clin. Psychol. Psychother. 27, 61–68. 10.1002/cpp.2407 31657075

[B13] Dobson J. (1997). MacIntyre's position on business: a response to Wicks. Business Ethics Quart. 7, 125–132. 10.2307/3857212

[B14] Drescher K. D. Foy D. W. Kelly C. Leshner A. Schutz K. Litz B. (2011). An explanation of the viability and usefulness of the construct of moral injury in war veterans. Traumatology 17, 8–13. 10.1177/1534765610395615

[B15] Farnsworth J. K. Drescher K. D. Evans W. Walser R. D. (2017). A functional approach to understanding and treating military-related moral injury. J. Context Behav. Sci. 6, 391–397. 10.1016/j.jcbs.2017.07.003

[B16] Fleming W. (2022). Complex moral injury: shattered moral assumptions. J. Relig. Health 61, 1022–1050. 10.1007/s10943-022-01542-4 35274226

[B17] Frankfurt H. (1999). Necessity, Volition, and Love. Cambridge: Cambridge University Press. 10.1017/CBO9780511624643

[B18] Gray M. J. Schorr Y. Nash W. Lebowitz L. Amidon A. Lansing A.. (2012). Adaptive disclosure: an open trial of a novel exposure-based intervention for service members with combat-related psychological stress injuries. Behav. Ther. 43, 407–415. 10.1016/j.beth.2011.09.001 22440075

[B19] Hodgson T. J. Carey L. B. (2017). Moral injury and definitional clarity: betrayal, spirituality and the role of chaplains. J. Relig. Health 56, 1212–1228. 10.1007/s10943-017-0407-z 28526912

[B20] Hodgson T. J. Carey L. B. (2019). “Moral injury within the RAAF and the role of chaplains: Exploratory findings,” in Conference Papers: Adelaide Convention Centre. Australian Military Medical Association Conference Papers, Vol. 27 (Journal of Military and Veterans Health), 58.

[B21] Holmes E. (2015). President Clinton Speaks with Elizabeth Holmes and Jack Ma [video file]. Available online at: https://www.youtube.com/watch?v=l7Be_6NEPQE (accessed July 28, 2022).

[B22] Jinkerson J. D. (2016). Defining and assessing moral injury: a syndrome perspective. Traumatology 22, 122–130. 10.1037/trm0000069

[B23] Knight K. (1998). The MacIntyre Reader. Notre Dame, IN: University of Notre Dame Press.

[B24] Koenig H. (2018). Measuring symptoms of moral injury in veterans and active duty military with PTSD. Religions 9, 86. 10.3390/rel903008631316405

[B25] Litz B. T. Stein N. Delaney E. Lebowitz L. Nash W. P. Silva C.. (2009). Moral injury and moral repair in war veterans: a preliminary model and intervention strategy. Clin. Psychol. Rev. 29, 695–706. 10.1016/j.cpr.2009.07.003 19683376

[B26] Lutz C. S. (2020). “Alasdair MacIntyre: An intellectual biography,” in Learning from MacIntyre, eds R. Beadle and G. Moore (Eugene: Pickwick Publications), 1–33.

[B27] MacIntyre (2016). Ethics in the Conflicts of Modernity: An Essay on Desire, Practical Reasoning, and Narrative. Cambridge: Cambridge University Press. 10.1017/9781316816967

[B28] MacIntyre A. ([1967] 1997). A Short History of Ethics: A History of Moral Philosophy From the Homeric Age to the Twentieth Century, 2nd Edn. Oxfordshire: Routledge. 10.2307/j.ctvpg85gr.

[B29] MacIntyre A. ([1981] 2007). *After Virtue, 3rd Edn*. London: Duckworth.

[B30] MacIntyre A. (1953). Marxism: An Interpretation. London: SCM Press.

[B31] MacIntyre A. (1958). Notes from the moral wilderness I. New Reasoner 7, 90–100.

[B32] MacIntyre A. (1959). Notes from the moral wilderness II. New Reasoner 8, 89–98.

[B33] MacIntyre A. (1973). Ideology, social science, and revolution. Comp. Polit. 5, 321–342. 10.2307/421268

[B34] MacIntyre A. (1999). Dependent Rational Animals: Why Human Beings Need the Virtues. Chicago, IL: Open Court.

[B35] Mantri S. Song Y. K. Lawson J. M. Berger E. J. Koenig H. G. (2021). Moral injury and burnout in health care professionals during the COVID-19 pandemic. J. Nerv. Ment. Dis. 209, 720–726. 10.1097/NMD.0000000000001367 34582400

[B36] Molendijk O. (2021). Moral Injury and Soldiers in Conflict: Political Practices and Public Perceptions. Oxfordshire: Routledge. 10.4324/9781003089957

[B37] Moore G. (2002). On the implications of the practice-institution distinction: MacIntyre and the application of modern virtue ethics to business. Business Ethics Quart. 12, 19–32. 10.2307/3857646

[B38] Moore G. (2005a). Humanizing business: a modern virtue ethics approach. Business Ethics Quart. 15, 237–255. 10.5840/beq200515212

[B39] Moore G. (2005b). Corporate character: modern virtue ethics and the virtuous corporation, Business Ethics Quart. 15, 659–685. 10.5840/beq200515446

[B40] Moore G. (2008). Re-imagining the morality of management: a modern virtue ethics approach. Business Ethics Quart. 18, 483–511. 10.5840/beq200818435

[B41] Moore G. (2012a). The virtue of governance: the governance of virtue. Business Ethics Quart. 22, 293–318. 10.5840/beq201222221

[B42] Moore G. (2012b). Virtue in business: alliance boots and an empirical exploration of MacIntyre's conceptual framework. Organiz. Stud. 33, 363–387. 10.1177/0170840611435599

[B43] Moore G. (2015). Corporate character, corporate virtues. Business Ethics Eur. Rev. 24, 99–114. 10.1111/beer.1210010126224

[B44] Moore G. (2016). “Corporate agency, character, purpose and the common good,” in The Challenges of Capitalism for Virtue Ethics and the Common Good: Inter-Disciplinary Perspectives, eds K. Akrivou and A. Sison (Cheltenham, PA: Edward Elgar), 159–165.

[B45] Moore G. (2017). Virtue at Work: Ethics for Individuals, Managers, and Organizations. Oxford: Oxford University Press. 10.1093/oso/9780198793441.001.0001

[B46] Moore G. Beadle R. (2006). In search of organizational virtue in business: agents, goods, practices, institutions, and environments. Organiz. Stud. 27, 369–389. 10.1177/0170840606062427

[B47] Nash W. P. Litz B. T. (2013). Moral injury: a mechanism for war-related psychological trauma in military family members. Clin. Child Family Psychol. Rev. 16, 365–337. 10.1007/s10567-013-0146-y 23852334

[B48] Potts G. (2020). The calling of the virtuous manager: politics shepherded by practical wisdom. Business Ethics Eur. Rev. 29, 6–16. 10.1111/beer.12300

[B49] Potts G. and Abadal, L.. (2023). “Moral injury and the spiritual roots of PTSD,” in Great Power Competition, Vol. 4 (Cham: Springer).

[B50] Richardson N. M. Lamson A. L. Smith M. Eagan S. M. Zvonkovic A. M. Jensen J. (2020). Defining moral injury among military populations: a systematic review. J. Trauma. Stress 33, 575–586. 10.1002/jts.22553 32567119

[B51] Robson A. (2015). Constancy and integrity: (un)measurable virtues? Business Ethics Eur. Rev. 24, 115–129. 10.1111/beer.12103

[B52] Sartre J. P. ([1947] 2007). Existentialism is a Humanism. Paris: Yale University Press. 10.12987/9780300242539.

[B53] Shay J. (1991). Learning about combat stress from Homer's *Iliad*. J. Trauma. Stress 4, 561–579. 10.1002/jts.2490040409

[B54] Shay J. (1994). Achilles in Vietnam: Combat Trauma and the Undoing of Character. Stoughton, WI: Simon and Schuster.

[B55] Shay J. (2002). Odysseus in America: Combat Trauma and the Trials of Homecoming. New York, NY: Scribner.

[B56] Shay J. (2011). Casualties. Daedalus 140, 179–188. 10.1162/DAED_a_00107 21898967

[B57] Shay J. (2014). Moral injury. Psychoanal. Psychol. 31, 182–191. 10.1037/a0036090

[B58] Sinnicks M. (2018). Leadership after virtue: MacIntyre's critique of management reconsidered. J. Business Ethics 147, 735–746. 10.1007/s10551-016-3381-6

[B59] Sison A. J. G. Redín D. M. (2022). If MacIntyre ran a business school… how practical wisdom can be developed in management education. Business Ethics Environ. Respons. 1–18. 10.1111/beer.12471

[B60] Stein N. R. Mills M. A. Arditte K. Mendoza C. Borah A. M. Resick P. A. (2012). A scheme for categorizing traumatic military events. Behav. Modif. 36, 787–807. 10.1177/0145445512446945 22679239

[B61] Williams R. Brundage J. Williams E. (2020). Moral injury in times of COVID-19. J. Health Serv. Psychol. 46, 65–69. 10.1007/s42843-020-00011-4 32363349 PMC7195905

